# Trigeminovascular calcitonin gene-related peptide release and peripheral vascular responses in a mouse model of accelerated aging: Implications for migraine

**DOI:** 10.1186/s10194-026-02441-9

**Published:** 2026-07-03

**Authors:** Linda Al-Hassany, Eloisa Rubio-Beltrán, Alejandro Labastida-Ramirez, Ingrid M. Garrelds, A. H. Jan Danser, Anton J. M. Roks, Antoinette MaassenVanDenBrink

**Affiliations:** 1 Division of Vascular Medicine and Pharmacology, Department of Internal Medicine, Erasmus MC, University Medical Center, PO Box 2040, Rotterdam, 3000 CA The Netherlands; 2 Present Address: Headache Group, Wolfson Sensory, Pain and Regeneration Centre, Institute of Psychiatry, Psychology and Neuroscience, King’s College London, London, UK; 3 Present Address: Division of Neuroscience, Faculty of Biology, Medicine, and Health, University of Manchester, Manchester, UK; 4 Geoffrey Jefferson Brain Research Centre, Manchester Academic Health Science Centre, Northern Care Alliance NHS Foundation Trust, University of Manchester, Manchester, UK

**Keywords:** Aging, CGRP, Mice, Migraine, Peripheral vasculature, Trigeminovascular system

## Abstract

**Objective:**

Understanding age-related changes in migraine is pivotal, considering the increasing global life expectancy. In addition, both aging and migraine are prominent cardiovascular risk factors. It remains unclear whether calcitonin gene-related peptide (CGRP) release changes with age across trigeminovascular components, and how this relates to peripheral responses to migraine-related vasodilatory molecules. The primary aim was to investigate age-related effects on CGRP release from the trigeminovascular system by studying a mouse model of combined accelerated neuronal and vascular aging, the DNA repair-deficient *Ercc1*^*Δ/−*^ mice. Second, we assessed the effects of aging on isolated coronary vasodilatory responses to CGRP and forskolin.

**Methods:**

Experiments were conducted using DNA repair-deficient *Ercc1*^*Δ/−*^mice and their wild type controls. After sacrifice, the trigeminal nucleus caudalis (TNC), trigeminal ganglion (TG), and dura mater (DM) were isolated. Ex vivo KCl-induced CGRP release was measured, and CGRP release was compared between *Ercc1*^*Δ/−*^ and wild type mice. In a subset of mice, concentration-response curves to CGRP and forskolin were generated in isolated coronary arteries. The pEC_50_ (negative log of the molar concentration of an agonist needed to reach half of its maximal effect) and E_max_ (maximum relaxation response) values were compared between both groups.

**Results:**

CGRP release (expressed as ratio compared to baseline release) of the DM was significantly lower in *Ercc1*^*Δ/−*^ (2.01 ± 0.24) *versus* wild type mice (3.22 ± 0.48) (P = 0.040). No differences were observed in CGRP release between *Ercc1*^*Δ/−*^and wild type mice in the TNC (8.07 ± 1.15 *versus* 6.10 ± 0.57, P = 0.364) or the TG (4.84 ± 0.92 *versus* 4.41 ± 0.60, P = 0.838). In addition, there were no differences in pEC_50_ and E_max_ values in response to CGRP and forskolin.

**Conclusion:**

Our findings suggest that aging is linked to reduced CGRP release of the DM, potentially partly explaining the reduction in migraine attacks in elderly. This decreased CGRP release is not accompanied by altered peripheral vascular reactivity to CGRP.

**Supplementary Information:**

The online version contains supplementary material available at 10.1186/s10194-026-02441-9.

## Plain Language summary

- Migraine headache is characterized by the release of a small protein called CGRP, which widens blood vessels and can trigger pain. However, it is not clear how this process is affected by aging.

- We studied a special type of mouse that ages faster to investigate whether this mouse released different amounts of CGRP in brain structures involved in migraine. Also, we tested how their heart blood vessels responded to CGRP and other molecules that also lead to widening of blood vessels.

- We found that older mice released less CGRP from a specific part of the nervous system, while their blood vessels responded similar to CGRP. This might explain why migraine attacks often become less frequent with increasing age.

**Supplementary Information:**

The online version contains supplementary material available at 10.1186/s10194-026-02441-9.

## Introduction

Migraine is a highly disabling and stigmatizing primary headache disorder that is characterized by unilateral moderate to severe headache attacks and associated symptoms (i.e., nausea, vomiting, photo- and phonophobia), which may be preceded or accompanied by aura symptoms in approximately one-third of patients [1–3]. The overall migraine prevalence is (at least) two-fold higher in women compared to men [4], while the migraine peak prevalence occurs at similar ages in both sexes – namely during the reproductive years (approximately 20s and 30s) with a second peak in women around fifty years of age [5]. Both the incidence and prevalence of migraine tend to decrease with advancing age [6, 7]. Understanding the neurovascular basis of these age-related changes is pivotal, especially considering the increase in global life expectancy [8].

The exact pathophysiological age-dependent mechanisms are not completely understood, but neuronal and vascular changes, besides hormonal senescence, have been hypothesized to be involved in migraine remission at older ages [6]. Activation of the trigeminovascular system and concomitant release of the vasodilatory neuropeptide calcitonin gene-related peptide (CGRP), expressed in both the central and peripheral components of the trigeminovascular system, play a causal role in the headache phase of migraine [9–12]. Interestingly, in rats, transport of CGRP in nerve fibers has been shown to decline significantly with advancing age [13]. In addition, in vivo studies have shown age-dependent changes in both pre- and post-synaptic peripheral fibers of these animals. These data indicate a reduction in the number of sensory fibers and/or a decrease in their peptide content or release, impacting the activation of the trigeminovascular system [14, 15]. Such a decline in CGRP content was also observed in specific brain structures, i.e., substantia nigra and striatum, as well as in cardiovascular tissue of aging rats [16] and in guinea pigs, whose maximum density of CGRP-containing perivascular nerve plexuses declined to approximately half in older age [17]. Being a neurovascular condition and an important cardiovascular risk factor [18], these alterations in trigeminal pain pathways should not be considered in isolation from the vascular age-related changes associated with migraine. Accordingly, previous research suggests that aging is associated with reduced cerebrovascular vasodilatory capacitance, probably accompanied by decreased activation of sensory neurons [6, 19] and disrupted neurovascular coupling [20].

It is yet unclear whether age-dependent differences in CGRP release exist across the central and peripheral structures of the trigeminovascular system. This knowledge is also relevant in the context of recently approved CGRP(-receptor)-targeting drugs in migraine, i.e., monoclonal antibodies and gepants, as their pharmacokinetic properties may be altered with aging [21]. Therefore, the primary aim of this study was to investigate the effects of aging on CGRP release from the trigeminal nucleus caudalis (central trigeminovascular component), trigeminal ganglion, and dura mater (both peripheral trigeminovascular components) by comparing CGRP release in a mouse model of accelerated aging, the DNA repair-deficient *Ercc1*^*Δ/−*^mice, *versus* their wild type littermates. The secondary objective was to deepen our understanding of the connection between neuronal and vascular age-related changes. We therefore studied vasodilatory responses of coronary arteries to CGRP and forskolin in *Ercc1*^*Δ/−*^ and wild type mice, as both lead to the accumulation of intracellular levels of cyclic adenosine monophosphate (cAMP) [22, 23], which, in turn, has been described to play a role in migraine induction [24, 25]. Further explorative analyses were conducted to evaluate the differences in trigeminovascular CGRP release between (i) male and female mice and (ii) the three components of the trigeminovascular system.

## Materials and methods

### Experimental mice

Experiments were performed using both male and female DNA repair-deficient *Ercc1*^*Δ/−*^ mice and their controls, i.e., wild type littermates (*Ercc1*^*+/+*^ mice). The *Ercc1*^*Δ/−*^ mice have one exon 7-truncated and one completely inactivated allele of the endonuclease *Ercc1*, thus partially inactivating this endonuclease that is involved in nucleotide excision and interstrand DNA crosslink repair. *Ercc1*^*Δ/−*^ mice with an F1 C57BL6J/FVB hybrid background were obtained, preventing strain-specific phenotypes [26, 27]. As previously described, *Ercc1*^*Δ/−*^ mice with a genetically uniform F1 C57BL6J/FVB hybrid background were obtained by crossing *Ercc1*^*Δ/+*^ mice (in a C57BL6J or FVB background) with *Ercc1*^*+/−*^ mice (in a FVB or C57BL6J background, respectively) [27], motivated by [28]. *Ercc1*^*Δ/−*^ mice represent a well-established model of accelerated neurovascular aging due to genomic instability, characterized by age-related diseases and a reduced lifespan (24–28 weeks compared to at least 100 weeks, and up to 146 weeks, in wild type mice). Overall, the accelerated aging phenotype includes neurodegeneration, osteoporosis, early-onset hypertension, vascular stiffness, accelerated endothelial and vasodilatory dysfunction, and cardiomyopathy [29–32], associated with an increase in phosphodiesterase 1 expression [33]. In addition, aortas from *Ercc1*^*Δ/−*^ mice exhibit phenotypic switching of vascular smooth muscle cells and an increased stress response [34]. Notably, a previous study on neuronal aging in these mice demonstrated that impaired DNA damage repair affects CGRP-labeled motor neurons [35]. However, while this murine model has not yet, to the best of our knowledge, been used in migraine-related research, it is useful for studying non-atherosclerotic vascular aging. This is relevant to migraine, especially since the known increased cardiovascular risk in migraine does not appear to be accompanied by large-vessel atherosclerosis [36, 37].

Breeding took place at the animal facility of the Erasmus Medical Center (Erasmus MC). Mice were kept in individually ventilated cages under controlled conditions (20–22 °C, 12-hour light/dark cycle) with free access to standard chow and water. All mice were weighed and visually inspected daily to monitor their well-being. The study consisted of three mice batches with identical genotypes, and all mice were sacrificed between 88 and 117 days of age. The study was planned and carried out in accordance with the Principles of Laboratory Animal Care and with guidelines approved by an independent Animal Ethics Committee, consulted at the Erasmus MC, as well as the ARRIVE guidelines.

### Calcitonin gene-related peptide release experiments in the trigeminal nucleus caudalis, trigeminal ganglion, and dura mater

The technique used to measure ex vivo CGRP release in the central (trigeminal nucleus caudalis) and peripheral (dura mater and trigeminal ganglion) components of the trigeminovascular system has been described previously [38, 39]. In short, mice were anesthetized using intraperitoneal sodium pentobarbital (80 mg/kg) and decapitated at the atlantooccipital joint. The skin and galea aponeurotica were both pulled back from the cranium. The trigeminal nucleus caudalis, located caudally between 9 and 13 mm from bregma, was first carefully isolated from the brainstem and divided into two parts. The skull was subsequently divided into two halves along the sagittal suture, and both cerebral hemispheres were removed, leaving the cranial dura mater intact and attached to the skull. The trigeminal ganglia of both sides were then obtained by dissecting 1 mm proximal and distal to the point where the mandibular nerve branches off. Thus, the skull was divided into two halves prior to removal of the trigeminal ganglia. The final step consisted of carefully hemisecting the cranium and removing the remaining (brain) tissue from the skull, except for the dura mater. For dura mater collection, only the portion attached to the middle cranial fossa was retained, while the remaining bone and tissue were removed to rule out the confounding effect of CGRP release from other extracranial structures. Each (half) of the isolated trigeminal nucleus caudalis, trigeminal ganglion, and skull with dura mater were completely immersed and washed in carbogenated synthetic interstitial fluid, containing NaCl (108 mM), KCl (3.48 mM), MgSO_4_ (3.5 mM), NaHCO_3_ (26 mM), NaH_2_PO_4_ (11.7 mM), CaCl_2_ (1.5 mM), sodium gluconate (9.6 mM), glucose (5.55 mM), and sucrose (7.6 mM) for three times 30 min at 37 °C to allow equilibration of the tissue samples, thus reducing false positive results due to manipulation of the tissue during preparation. Notably, while this washing time is in line with previous experiments, a possible drawback is the risk of compromising tissue integrity.

Both halves of the isolated tissues were then placed in a 24-well plate containing 500 µL of synthetic interstitial fluid per well. The plate was fixed in a water bath, creating a closed, humid and carbogenated chamber of 37 °C. Tissues were washed three additional times over a period of up to 15 min. Basal CGRP levels were measured in each tissue, after which CGRP release was induced by adding 60 mM KCl, a submaximal depolarizing stimulus of which the use and concentration are in line with previous (rodent) studies on CGRP release in the trigeminovascular system [38–41]. Both the baseline and KCl-infused samples were collected after 10 min of incubation and mixed with aprotinin (500 KIU/mL).

Samples were stored at − 80 °C until CGRP content was assessed using a commercial CGRP RIA kit according to the manual (Phoenix Pharmaceuticals, Burlingame, CA, USA). The assay has a detection level of 1.0 pg/mL; samples with CGRP concentrations below this limit were assigned this value of 1.0 pg/mL, in line with our previous study [38]. Blanks containing only synthetic interstitial fluid, without CGRP, were used as a control to exclude false-positive measurements. For the CGRP assays, the analyst was blinded to the mice’s genotype. CGRP release was assessed in duplicate for both individual halves of each trigeminovascular structure, of which averages were used for further analyses. CGRP release was expressed as the ratio of KCl-induced CGRP release to basal CGRP release, which represents the relative stimulated release of CGRP (i.e., CGRP release ratio). Tissues that exhibited either no or decreased relative CGRP release in response to 60 mM KCl (i.e., CGRP release ratios ≤ 1.05) or an unusually high relative release (i.e., CGRP release ratios ≥ 100) were excluded from further analyses, consistent with our previous study on CGRP release in mice [39]. For each half of each trigeminovascular structure, the CGRP release ratio was calculated separately, after which both values were averaged to obtain one value per animal. In case where exclusion criteria applied, only one tissue sample, or none, was included for that animal. As the ex vivo experiments consisted of three mouse batches, an outlier test – i.e., the ROUT method (Q = 1%) – was consequently performed on each individual batch and the combined data of all batches.

CGRP release experiments were performed on a total of 27 *Ercc1*^*Δ/−*^ mice and 29 wild type mice, of whom tissues were obtained and analyzed from both halves. From 1 *Ercc1*^*Δ/−*^ mouse, no trigeminal nucleus caudalis and trigeminal ganglion were obtained due to damaged tissue.

### Peripheral vasodilatory experiments in coronary arteries

Immediately after sacrificing the mice, coronary arteries were carefully isolated in oxygenated and carbogenated Krebs buffer solution (NaCl 118 mM, KCl 4.7 mM, CaCl_2_ 2.5 mM, MgSO_4_ 1.2 mM, KH_2_PO_4_ 1.2 mM, NaHCO_3_ 25 mM, and glucose 8.3 mM in distilled water, pH 7.4).

Functional experiments on the coronaries were conducted only for 6 wild type and 6 *Ercc1*^*Δ/−*^ mice concurrently with the CGRP release experiments. For these experiments, one vessel segment of 2 mm length per mouse was mounted in small wire Mulvany myograph organ baths (Danish Myograph Technology, Aarhus, Denmark), containing 6 mL of oxygenated and carbogenated Krebs solution at 37 °C. The mounted vessel segments were first left to equilibrate before the tension was normalized by stepwise stretching vessels to a tension corresponding to 90% of the estimated diameter at a transmural pressure of 100 mmHg [42]. After four washing steps, each separated by a 5-minute interval, maximum contractile responses were determined using 60 mmol/L KCl as a reference contraction. To evaluate vasodilatory responses, coronary segments were precontracted with 30 nmol/L of U46619 (a thromboxane A_2_ analog) to study the effects of forskolin and with 30 mmol/L of KCl (facilitating comparison with human data from our lab, where precontractions are also induced by KCl) to study the effects of CGRP. After reaching a contraction plateau, concentration-response curves were sequentially generated in a single segment for the following vasodilators using cumulative doses: CGRP (10^− 11^–10^− 6^ mol/L in half-logarithmic steps) and forskolin (10^− 9^–10^− 4^ mol/L in half-logarithmic steps).

Data were recorded using LabChart data acquisition software (AD Instruments Ltd, Oxford, UK). Relaxation responses to the vasodilatory substances are expressed as percentages (%) relative to the contraction induced by either 30 nmol/L U46619 or 30 mmol/L KCl, which was set at 100%. Concentration-response curves with a sigmoidal shape were constructed using a computerized curve-fitting technique, in which the bottom was constrained to zero. Non-linear regression analysis was used to determine the pEC_50_ (negative log of the molar concentration of an agonist needed to reach half of its maximal effect) and E_max_ (maximum relaxation response) values.

### Statistical analyses

CGRP release ratios, the baseline CGRP release, and percentages of relaxation are all expressed as mean ± SEM. Age is expressed as median [Q_1_–Q_3_]. Statistical differences in the relative CGRP release, pEC_50_ and E_max_ values between wild type and *Ercc1*^*Δ/−*^ mice were calculated using the two-tailed Mann-Whitney test for unpaired observations. A nonparametric test was applied to compare differences in CGRP release, in accordance with previous studies [38, 39], and due to the small sample size of vasodilatory responses in coronary arteries. To additionally explore differences between male and female wild type and *Ercc1*^*Δ/−*^ mice, a Kruskal-Wallis test with a post-hoc test, corrected Dunn’s multiple comparisons tests, was applied. Similarly, differences in CGRP release between the trigeminal nucleus caudalis, trigeminal ganglion, and dura mater were analyzed using the Kruskal-Wallis test with Dunn’s multiple comparisons test, stratified by wild type and *Ercc1*^*Δ/−*^ mice.

The statistical significance level was set at *P* ≤ 0.05. All analyses were performed using GraphPad Prism version 8.0.1 for Windows (GraphPad Software, Boston, Massachusetts USA).

### Compounds

Forskolin was obtained from Tocris BioScience (Bristol, UK) and dissolved in physiological saline. U46619 was purchased from Sigma Chemical Co. (St. Louis, MO, USA); r-α-CGRP was obtained from PolyPeptide (Strasbourg, France).

## Results

### Calcitonin gene-related peptide release in the trigeminal nucleus caudalis, trigeminal ganglion, and dura mater

#### Trigeminal nucleus caudalis

**Fig. 1:**
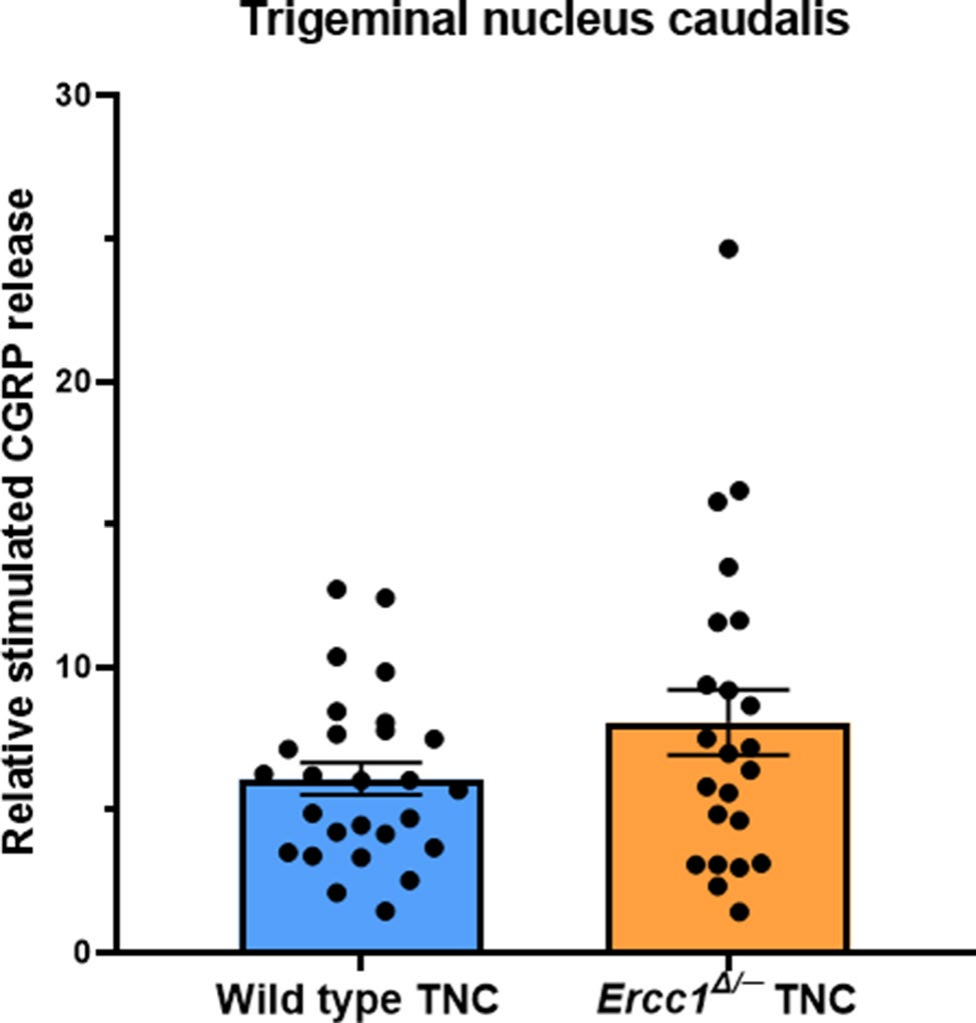
Bar plots with the mean ± SEM of the relative stimulated calcitonin gene-related peptide (CGRP) release ratios of the trigeminal nucleus caudalis (TNC) after potassium chloride (KCl) administration in wild type (*n* = 27) and *Ercc1*^*Δ/−*^ mice (*n* = 23) (*P* > 0.05)

Exclusion of individual tissues of the trigeminal nucleus caudalis based on the previously mentioned criteria of the CGRP release ratio and outlier tests yielded averaged ratios from 23 *Ercc1*^*Δ/−*^ mice and 27 wild type mice. The *Ercc1*^*Δ/−*^ mice consisted of 9 males (39%) and 14 females (61%) with a median age of 92 [90–111] days. The wild type mice consisted of 14 males (52%) and 13 females (48%) with a median age of 110 [96–114] days. Basal CGRP release (i.e., prior to stimulation with KCl) of the trigeminal nucleus caudalis was 83.7 pg/mL ± 11.6 for *Ercc1*^*Δ/−*^ mice and 134.9 pg/mL ± 20.6 for wild type mice. No significant differences were observed in CGRP release of the trigeminal nucleus caudalis between *Ercc1*^*Δ/−*^ mice (8.07 ± 1.15) and wild type mice (6.10 ± 0.57) (*P* = 0.364), Fig. 1.

#### Trigeminal ganglion

**Fig. 2:**
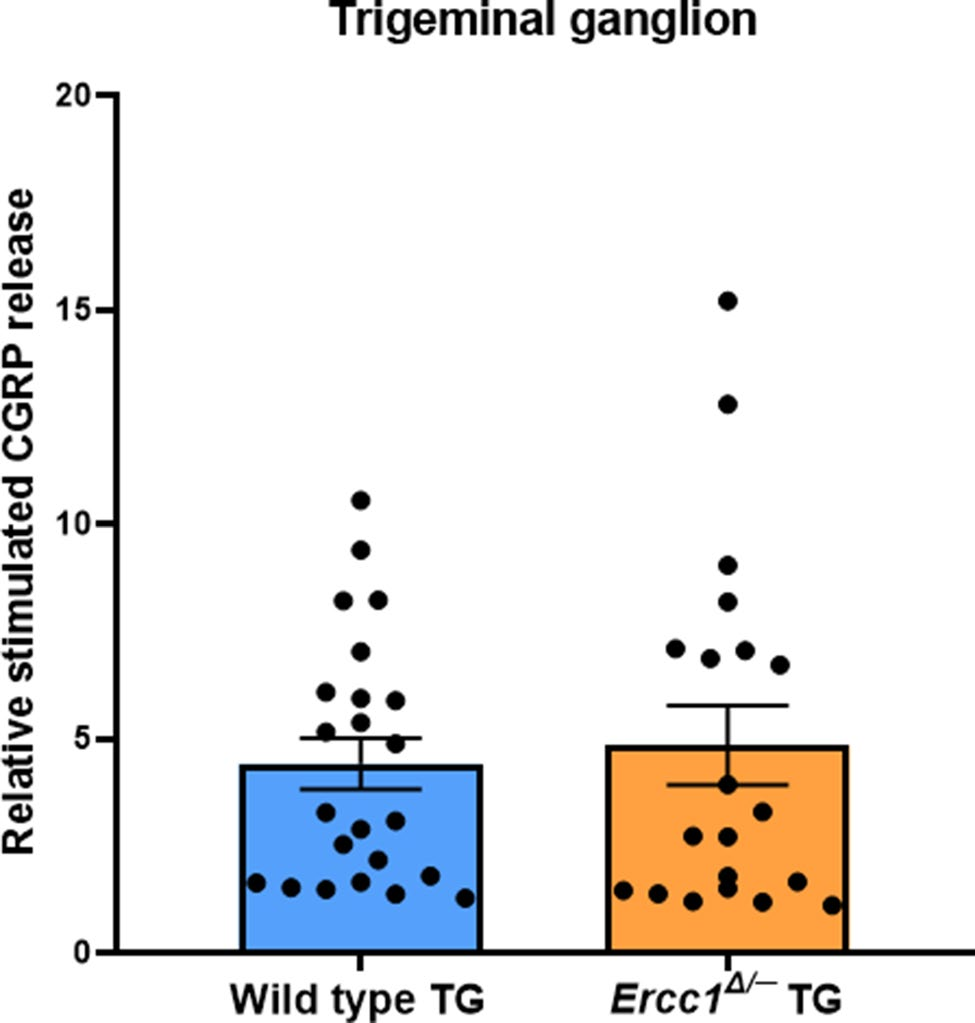
Bar plots with the mean ± SEM of the relative stimulated calcitonin gene-related peptide (CGRP) release ratios of the trigeminal ganglion (TG) after potassium chloride (KCl) administration in wild type (*n* = 23) and *Ercc1*^*Δ/−*^ mice (*n* = 20) (*P* > 0.05)

Exclusion of individual tissues of the trigeminal ganglion based on the previously mentioned criteria yielded averaged ratios from 20 *Ercc1*^*Δ/−*^ mice and 23 wild type mice. The *Ercc1*^*Δ/−*^ mice consisted of 10 males (50%) and 10 females (50%) with a median [Q_1_–Q_3_] age of 91 [90–106.8] days. The wild type mice consisted of 11 males (48%) and 12 females (52%) with a median age of 110 [92–114] days. Basal CGRP release (i.e., prior to stimulation with KCl) of the trigeminal ganglion was 19.8 pg/mL ± 7.4 for *Ercc1*^*Δ/−*^ mice and 8.9 pg/mL ± 1.8 for wild type mice. No significant differences were observed in CGRP release of the trigeminal ganglion between *Ercc1*^*Δ/−*^ mice (4.84 ± 0.92) and wild type mice (4.41 ± 0.60) (*P* = 0.838), Fig. 2.

#### Dura mater

**Fig. 3:**
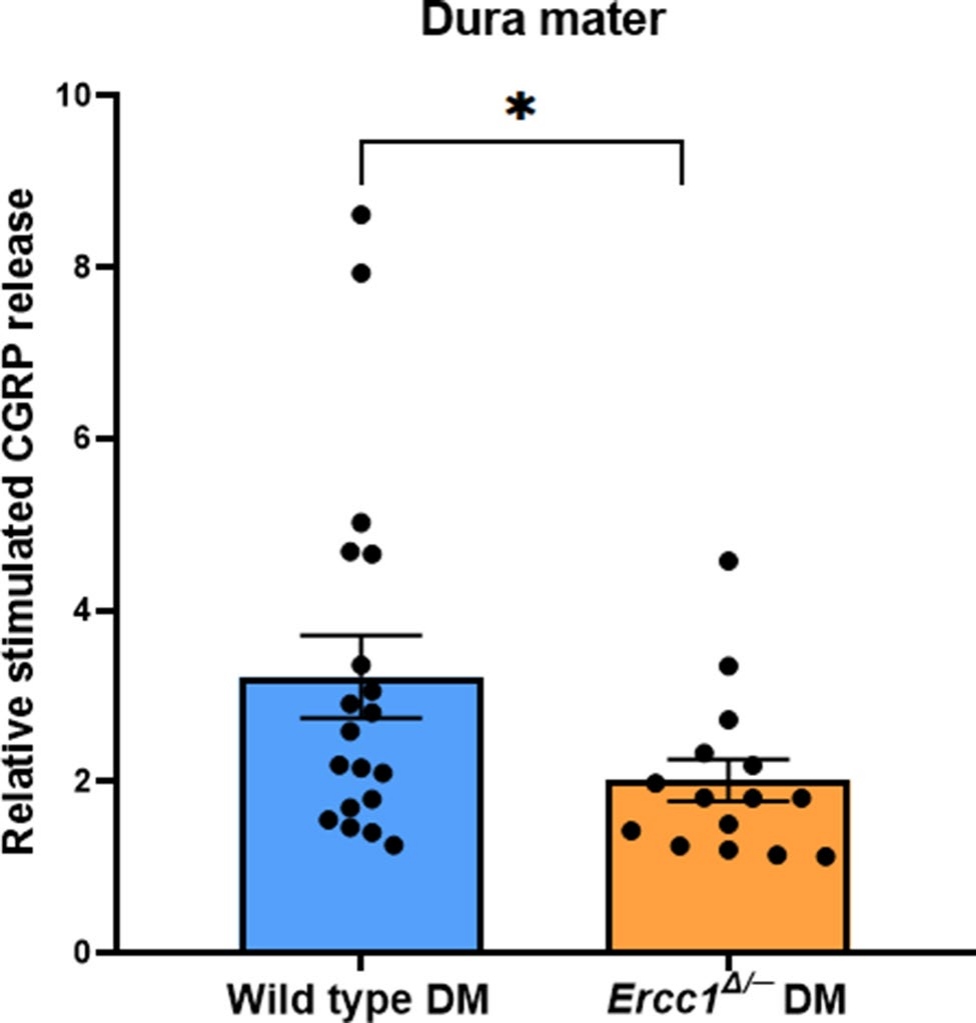
Bar plots with the mean ± SEM of the relative stimulated calcitonin gene-related peptide (CGRP) release ratios of the dura mater after potassium chloride (KCl) administration in wild type (*n* = 19) and *Ercc1*^*Δ/−*^ mice (*n* = 15) (* indicates *P* < 0.05)

Exclusion of individual tissues of the dura mater based on the previously mentioned criteria yielded averaged ratios from 15 *Ercc1*^*Δ/−*^ mice and 19 wild type mice. The *Ercc1*^*Δ/−*^ mice consisted of 8 males (53%) and 7 females (47%) with a median [Q_1_–Q_3_] age of 92 [90–112] days. The wild type mice consisted of 11 males (58%) and 8 females (42%) with a median age of 112 [95–114] days. Basal CGRP release (i.e., prior to stimulation with KCl) of the dura mater was 44.7 pg/mL ± 16.4 for *Ercc1*^*Δ/−*^ mice and 14.2 pg/mL ± 3.3 for wild type mice. Significantly lower CGRP release of the dura mater were observed in *Ercc1*^*Δ/−*^ mice (2.01 ± 0.24) compared to wild type mice (3.22 ± 0.48) (*P* = 0.040), Fig. 3.

#### Analyses on male-female differences and different trigeminovascular system components

We further explored differences in CGRP release between male and female *Ercc1*^*Δ/−*^ mice and wild type mice. While the overall Kruskal-Wallis test was significant (*P* < 0.0001), we observed no differences between males and females of the trigeminal nucleus caudalis, trigeminal ganglion, and dura mater of both *Ercc1*^*Δ/−*^ and wild type mice (Supplemental Fig. 1). However, significant differences were observed among components of the trigeminovascular system in both groups of mice. In both, *Ercc1*^*Δ/−*^ mice (overall Kruskal-Wallis test *P* < 0.0001) and wild type mice (overall Kruskal-Wallis test *P* = 0.002), CGRP release was the highest in the trigeminal nucleus caudalis, followed by the trigeminal ganglion, and the lowest in the dura mater. In *Ercc1*^*Δ/−*^ mice, post-hoc testing indicated significant differences between the trigeminal nucleus caudalis and trigeminal ganglion (adjusted *P* = 0.036) and the dura mater (adjusted *P* < 0.0001), but not between the trigeminal ganglion and dura mater (adjusted *P* = 0.146), Supplemental Fig. 2. In wild type mice, post-hoc testing indicated only significant differences between the trigeminal nucleus caudalis and dura mater (adjusted *P* = 0.002) but not between the trigeminal nucleus caudalis and trigeminal ganglion (adjusted *P* = 0.093) nor between the trigeminal ganglion and dura mater (adjusted *P* = 0.575), Supplemental Fig. 3.

### Peripheral vasodilatory responses in coronary arteries

Coronary arteries were obtained from a total of 6 *Ercc1*^*Δ/−*^ mice (aged 90 [90–90] days, including 2 females) and 6 wild type mice (aged 91.5 [90.5–92.3] days, including 2 females). Responses to 30 mmol/L KCl resulted in a significantly lower mean contraction of 0.72 ± 0.13 mN in *Ercc1*^*Δ/−*^ mice and 1.91 ± 0.21 mN in wild type mice (*P* = 0.002). Similarly, responses to 30 nmol/L of U46619 resulted in a significantly lower mean contraction of 1.10 ± 0.21 mN in *Ercc1*^*Δ/−*^ mice and 2.63 ± 0.24 mN in wild type mice (*P* = 0.004), Supplemental Figs. 4 and 5.

**Fig. 4:**
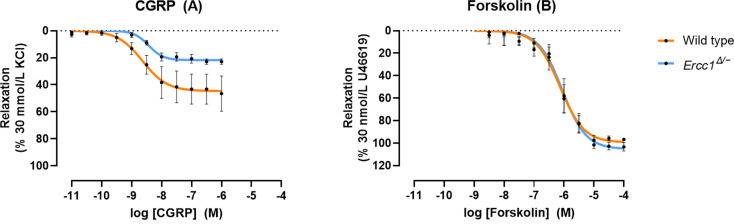
Concentration-response curve showing relaxation in response to calcitonin-gene related peptide (CGRP) (**A**) and forskolin (**B**), relative to either potassium chloride (KCl) or U46619, in coronary arteries of wild type mice (*n* = 6) and *Ercc1*^*Δ/−*^ mice (*n* = 6)

Concentration-response curves of vasodilatory responses to CGRP and forskolin in coronaries obtained from wild type and *Ercc1*^*Δ/−*^ mice are depicted in Fig. 4. Corresponding pEC_50_ and E_max_ values are presented for both mice groups in Table 1. The concentration-response curves to CGRP and forskolin showed no differences in the potency, nor in the maximum response, between *Ercc1*^*Δ/−*^ and wild type mice.

**Table 1:** Vasodilatory responses to calcitonin-gene related peptide (CGRP) and forskolin

Substance	pEC_50_			E_max_ (%)		
			*P*-value			*P*-value
	*Ercc1* ^Δ/−^	*Wild type*		*Ercc1* ^Δ/−^	*Wild type*	
CGRP	8.00 ± 0.42	8.63 ± 0.08	0.193	22.86 ± 1.85	43.23 ± 11.04	0.132
Forskolin	6.07 ± 0.18	6.07 ± 0.16	> 0.999	102.68 ± 3.03	99.39 ± 2.79	0.132

The mean ± SEM of the corresponding pEC_50_ (i.e., negative log of the molar concentration of an agonist needed to reach half of its maximal effect) and average E_max_ (maximum relaxation response) values are presented and compared between both wild type mice (*n* = 6) and *Ercc1*^*Δ/−*^ mice (*n* = 6). An asterisk (*) indicates *P* < 0.05

## Discussion

In this study, the effects of aging on trigeminovascular CGRP release and peripheral vascular responses of coronaries were investigated, comparing DNA repair-deficient *Ercc1*^*Δ/−*^ mice to their controls. We observed reduced CGRP release of the dura mater in *Ercc1*^*Δ/−*^ mice compared to wild type mice. No differences were observed in the trigeminal ganglion or in the central part of the trigeminovascular system, i.e., the trigeminal nucleus caudalis.

To the best of our knowledge, no previous studies have focused on the effects of aging on CGRP release in the trigeminovascular system. Admittedly, Bergman and colleagues observed an age-related reduction in CGRP expression, both at the cellular peptide level and mRNA level, in primary sensory neurons of the cervical and lumbar dorsal root ganglia of aged rats compared to young adults [43]. These processes are hypothesized to be caused by aging-related lesions of axons. Our results might suggest that aging selectively affects peripheral nociceptive nerves in the dura mater rather than the entire trigeminovascular system – hinting towards a reduction in the density or functional integrity of nociceptive CGRP-positive nerve fibers in this structure. Indeed, previous preclinical studies in rats have shown a decrease in axoplasmic transport of CGRP in peripheral nerves [13] and changes in sensory nerve function, including impaired neuropeptide release from peripheral terminals, with advancing age [14]. Rodent studies have also confirmed an age-related decline in CGRP in the peripheral vascular system. Further, immunoreactive CGRP levels were observed to be lower in both plasma and mesenteric (resistance) arteries of middle-aged female rats compared to young adult rats [44], and a decline in aortic CGRP-positive neuronal fibers was observed during aging [45]. An age-dependent decline in CGRP concentrations has also been observed in human middle cerebral arteries [46]. Our results on the dura mater confirm a previous study by De Vries et al. on age- and sex-dependent CGRP-induced vasodilation in human isolated vessels, distinguishing responses in both younger and older men and women [47]. While the latter study did not focus on CGRP release, age-related differences were observed in middle meningeal arteries, with a negative correlation between the maximum response to CGRP with age in men. Whether this is caused by the decreased release in elderly, potentially leading to receptor downregulation, remains to be demonstrated.

In our study, the dura mater exhibited the lowest levels of CGRP release compared to the trigeminal ganglion and trigeminal nucleus caudalis in both *Ercc1*^*Δ/−*^ and wild type mice. A previous rodent study showed that in the dura mater, CGRP is expressed in unmyelinated C-fibers, probably acting on CLR and RAMP1-expressing myelinated Aδ-fibers – of which an age-related decline has been previously observed [48] – mast cells, and vascular smooth muscle cells [11]. Another study found no expression of these CGRP-receptor components on sensory axons in the cranial dura mater [49]. It should be acknowledged that the applied hemiskull model in mice is relatively fragile, potentially resulting in large variability between mice and a relatively greater exclusion of CGRP release ratios of the dura mater; many research groups therefore favor the rat model instead [50, 51]. However, we were confined to the use of these mice, which allowed us to investigate the effects of neurovascular aging.

We did not study the meningeal artery in the myograph because the dura mater had already been used for the aforementioned experiments. Therefore, we studied the coronary artery to assess potential peripheral responses and observed no significant differences in responses to CGRP and forskolin. While this is consistent with human data showing no differences in functional responses to CGRP in young and aged human coronary arteries [47], we cannot categorically exclude that the small number of coronary experiments limited the statistical power of our study, given the trend we observed for diminished CGRP responses in the *Ercc1*^*Δ/−*^ animals. If this would indeed be the case, as would be in accordance with other mouse studies [52], this would most likely involve a mechanism independent of downstream alterations in the smooth muscle cAMP signaling pathway, as responses to forskolin were identical between groups in our study, which is in accordance with a previous study showing no age-related changes in adenylate cyclase activity in rat aorta [53]. In contrast, a potentially decreased response to CGRP could involve endothelial and/or cyclic guanosine monophosphate (cGMP)-dependent mechanisms, which we previously showed to be affected in these *Ercc1*^*Δ/−*^ mice [54]. Indeed, while CGRP-induced relaxations in human coronary arteries are endothelium-independent [55], endothelium-dependent mechanisms seem to be involved in mice [56] and in rodent arteries precontracted with noradrenaline [57]. Notably, a previous study using isolated aortic and coronary segments from *Ercc1*^*Δ/−*^mice also demonstrated impaired vasomotor responses to acetylcholine and sodium nitroprusside, indicating alterations in endothelium-dependent and endothelium-independent relaxations [54]. Thus, while our findings hint towards a possibility of altered CGRP-mediated vasodilation, the available evidence does not establish this effect as CGRP-specific, given the contribution of broader age-related vascular dysfunction.

Lastly, we compared trigeminovascular CGRP release between male and female mice and observed no differences. As the menstrual cycle is known to influence the CGRP-mediated trigeminovascular responsiveness in human experiments [58, 59], it is important to note that both male and female *Ercc1*^*Δ/−*^ mice were previously shown to be infertile [60], and thus, sex differences may have been obscured.

### Clinical translation: Implications for migraine and future research

The vascular dilatory capacity decreases with age, while the frequency and severity of migraine decline with age as well [6]. However, no conclusive evidence exists on the exact neuronal and vascular age-related changes in migraine. While direct comparisons regarding CGRP release or activity cannot be made, our findings in the current study are in accordance with our previous study in humans, showing that capsaicin-induced trigeminal nerve-mediated vasodilation at the forehead was significantly reduced in postmenopausal women compared to younger women, while peripheral microvascular reactivity was preserved and comparable to that of younger women [59].

Our findings might partly explain the reduction in migraine headache attacks in elderly, considering the important meningeal contribution to migraine pain. Indeed, the dura mater is pain-sensitive in proximity to the meningeal arteries, related to the activation of dural perivascular nociceptors [61]. Release of neuropeptides leads to vasodilation and degranulation of mast cells in the dura mater in animal models [12]. In this context, it is tempting to hypothesize that the previously described reduction in migraine headache with advancing age [62] may relate to diminished dural CGRP release, possibly acting alongside age-related vascular stiffening in patients where migraine declines during lifetime [63, 64]. In contrast, migraine aura symptoms may persist into older age due to central mechanisms [62] – potentially reflected by unaltered CGRP release in the central structures of the trigeminovascular system as observed in our study – that are less affected by age. However, while we currently lack evidence to confirm or refute this hypothesis, future studies should address these peripheral and central age-dependent effects, including the influence on cortical spreading depression – a key mechanism underlying migraine aura [65]. In our study, the decreased trigeminovascular CGRP release from the dura mater in *Ercc1*^*Δ/−*^mice did not seem to be accompanied by an altered peripheral vascular reactivity to CGRP, although no conclusions regarding peripheral CGRP release can be drawn. Notably, we did not investigate the release of other neuropeptides, such as adrenomedullin and amylin, which often colocalize with CGRP, in the trigeminovascular system [66, 67]. Importantly, however, these findings do not establish Ercc1^Δ/−^ mice as a model of migraine in aging, but rather suggest that aging may affect trigeminovascular CGRP release and CGRP-associated vascular mechanisms relevant to migraine biology.

Given that aging is one of the most significant risk factors for cardiovascular disease, that migraine is increasingly recognized as a cardiovascular risk factor, and that CGRP plays a key role in cardiovascular health and vascular aging processes [68–70], our results offer new pathophysiological insights into the involvement of specific vasodilatory mechanisms herein.

## Conclusion

Taken together, this study demonstrates a reduction in CGRP release from the dura mater – but not from the trigeminal nucleus caudalis or the trigeminal ganglion – in a mouse model of accelerated aging. No differences in peripheral vasodilatory responses to CGRP and forskolin were found, suggesting that reduced trigeminovascular CGRP release is not associated with altered peripheral vascular responsiveness to CGRP in older mice – a finding which may, however, differ for other peptides and therefore warrants further research. An age-related decline in the density or functional integrity of nociceptive CGRP-positive nerve fibers in the dura mater may, at least partly, explain the reduction in the frequency of migraine headache attacks in elderly.

## Supplementary Information

Below is the link to the electronic supplementary material.

Supplementary Material 1

## Data Availability

Data are available upon reasonable request to the corresponding author.
