# Supplementary material for: Trigeminovascular calcitonin gene-related peptide release and peripheral vascular responses in a mouse model of accelerated aging: Implications for migraine
Source: J Headache Pain. 2026 Jul 3;27(1):169. doi: 10.1186/s10194-026-02441-9 (PMC13340349; doi:10.1186/s10194-026-02441-9)
Supplement: Supplementary file 1 — Supplementary Material 1 [file 10194_2026_2441_MOESM1_ESM.docx]

# **SUPPLEMENTARY MATERIAL**


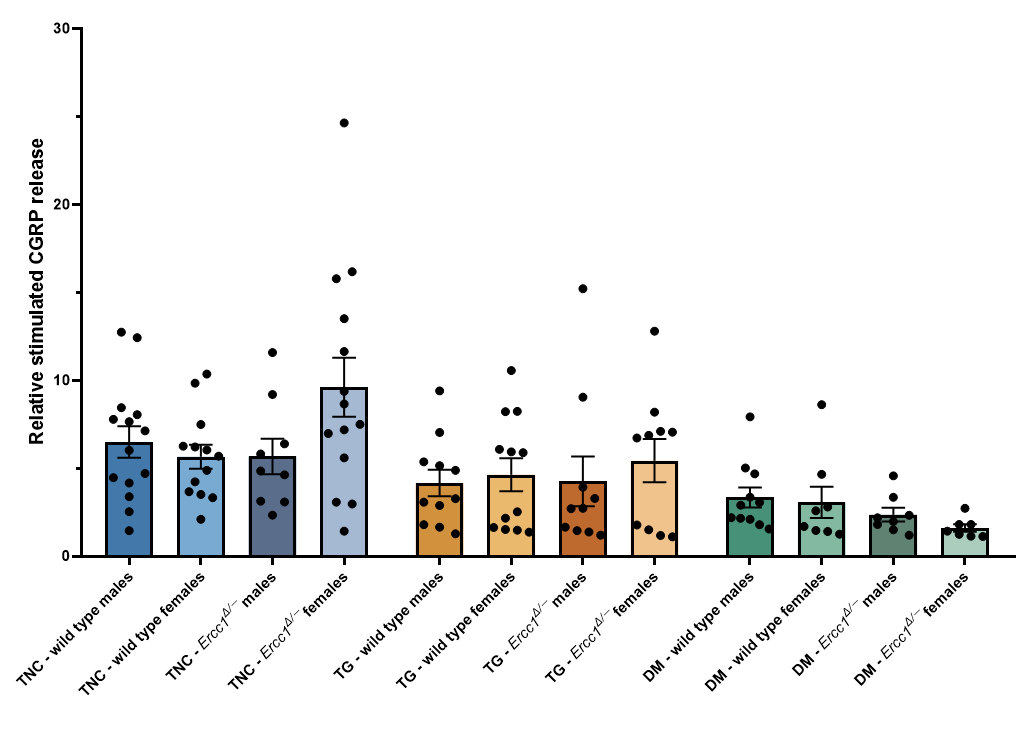


**Supplemental figure 1** – Bar plots with the mean ± SEM of the sex-stratified relative stimulated calcitonin gene-related peptide (CGRP) release ratios of the trigeminal nucleus caudalis (TNC), trigeminal ganglion (TG), and dura mater (DM) after potassium chloride (KCl) administration in both wild type and *Ercc1^Δ/−^* mice, indicating no significant differences between males *versus* females for the TNC (adjusted *P*>0.999 for wild type mice and adjusted *P*>0.999 for *Ercc1^Δ/−^* mice), TG (adjusted *P*>0.999 for wild type mice and adjusted *P*>0.999 for *Ercc1^Δ/−^* mice), nor the DM (adjusted *P*>0.999 for wild type mice and adjusted *P*>0.999 for *Ercc1^Δ/−^* mice).


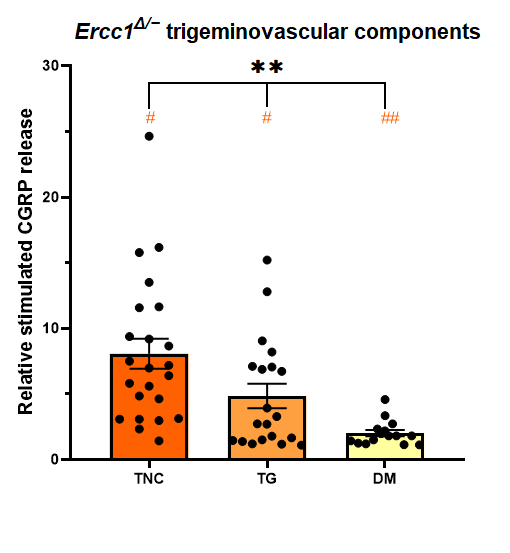


**Supplemental figure 2** – Bar plots with the mean ± SEM of the relative stimulated calcitonin gene-related peptide (CGRP) release ratios of the trigeminal nucleus caudalis (TNC, n = 23), trigeminal ganglion (TG, n = 20), and dura mater (DM, n = 15) after potassium chloride (KCl) administration in *Ercc1^Δ/−^* mice, indicating overall significant differences between the three groups (****** indicates overall *P*< 0.0001).

Post-hoc tests indicated significant differences between TNC *versus* TG and TNC *versus* DM, but not between TG *versus* DM. # indicates statistical significance with an adjusted *P*< 0.05 of TNC *versus* TG, while ## indicates statistical significance with an adjusted *P*< 0.0001 of TNC *versus* DM.


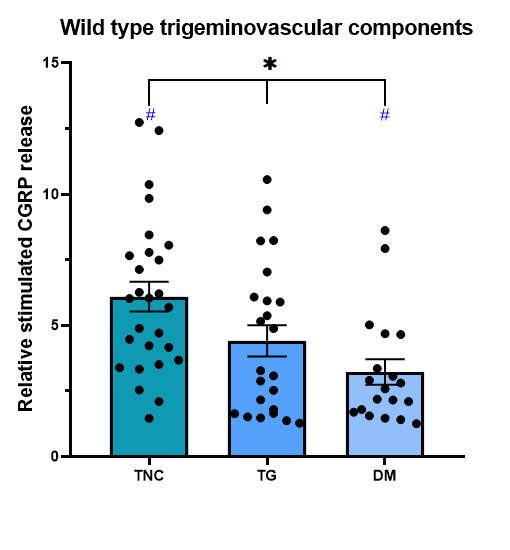


**Supplemental figure 3** – Bar plots with the mean ± SEM of the relative stimulated calcitonin gene-related peptide (CGRP) release ratios of the trigeminal nucleus caudalis (TNC, n = 27), trigeminal ganglion (TG, n = 23), and dura mater (DM, n = 19) after potassium chloride (KCl) administration in wild type mice, indicating overall significant differences between the three groups (***** indicates overall *P*< 0.05).

Post-hoc tests indicated significant differences between TNC *versus* DM, but not between TNC *versus* TG nor between TG *versus* DM. # indicates statistical significance with an adjusted *P*< 0.05.


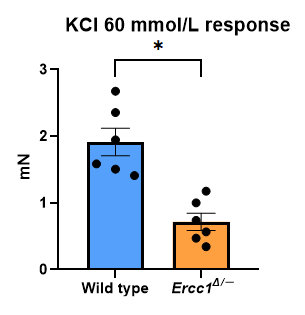


**Supplemental figure 4** – Bar plot with the mean ± SEM contractile responses to 60 mmol/L potassium chloride (KCl) of coronaries in wild type (n = 6) and *Ercc1^Δ/−^* mice (n = 6). An asterisk (*****) indicates a *P*< 0.05).


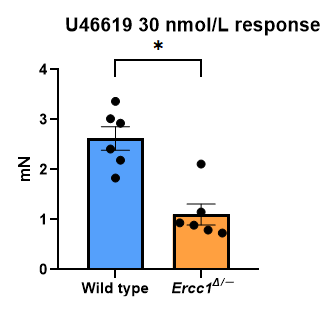


**Supplemental figure 5** – Bar plot with the mean ± SEM contractile responses to 30 nmol/L U46619 of coronaries in wild type (n = 6) and *Ercc1^Δ/−^* mice (n = 6). An asterisk (*****) indicates a *P*< 0.05).
